# B_*x*_C_*y*_N_*z*_ hybrid graphenylene: stability and electronic properties†

**DOI:** 10.1039/c8ra02188k

**Published:** 2018-07-10

**Authors:** A. Freitas, L. D. Machado, C. G. Bezerra, R. M. Tromer, L. F. C. Pereira, S. Azevedo

**Affiliations:** Departamento de Física, Universidade Federal do Rio Grande do Norte 59072-970 Natal RN Brazil alilianefisica@yahoo.com.br; Departamento de Física, CCEN, Universidade Federal da Paraíba Caixa Postal 5008 58051-970 João Pessoa PB Brazil

## Abstract

Interest in hybrid monolayers with arrangements that differ from that of the honeycomb lattice has been growing. However, systematic investigations on the properties of these structures are still lacking. In this work, we combined density functional theory (DFT) and molecular dynamics (MD) simulations to study the stability and electronic properties of nanosheets composed of B, C, and N atoms arranged in the pattern of the carbon allotrope graphenylene. We considered twenty structures with varied atomic arrangements and stoichiometries, which we call B_*x*_C_*y*_N_*z*_ hybrid graphenylenes. We calculated the formation energy for each arrangement, and found that it decreases as the number of B–C and N–C bonds decreases. We also found that the structure with minimum energy has stoichiometry B_2_CN and an atomic arrangement with BN and C stripes connected along the zigzag direction. Regarding the electronic properties, we found that all investigated structures are semiconductors, with band gaps ranging from 0.14 to 1.65 eV. Finally, we found that the optimized hybrid lattices presented pores of varied sizes and shapes. This diversity in pore geometry suggests that these structures might be particularly suited for molecular sieve applications.

## Abstract 2

We combine density functional theory and molecular dynamics to investigate the stability and electronic properties of 20 structures composed of boron, carbon, and nitrogen arranged in the pattern of the two-dimensional carbon allotrope graphenylene.

## Graphical abstract

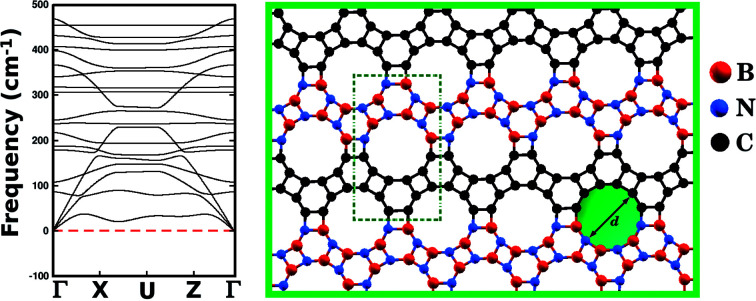

## 1. Introduction

Over the last years two-dimensional (2D) materials have attracted considerable interest, especially those composed of sp^2^-hybridized atoms arranged in a honeycomb lattice, due to their novel properties and potential applications in many emerging technologies.^1^ Examples of already synthesized 2D materials include graphene and hexagonal boron-nitride (h-BN).^2–5^ Graphene is a zero-band-gap semiconductor, with high electron mobility at room temperature, high thermal conductivity, absorbs 2.3% of incident white light, and has mechanical properties that characterize it as a strong and flexible material.^6–10^ On the other hand, h-BN is a large-band-gap semiconductor (>4 eV), with mechanical properties similar to graphene, and exhibits high thermal and chemical stability.^11–13^ In this context, an interesting possibility consists in combining the properties of these two materials by replacing C atoms in the graphene lattice by B and N atoms, leading to the formation of B_*x*_C_*y*_N_*z*_ hybrid monolayers,^14–19^ some of which have recently been synthesized.^20,21^ These structures typically present small energy gaps (<2 eV), which are intermediate between those found for graphene and h-BN. However, by tuning the atomic arrangements and stoichiometries (*x*, *y*, *z*), they can also present metallic and semi-metallic behavior.^22–24^ Due to this high diversity of energy band gaps, these materials have been regarded as ideal candidates for the next generation of nanoscale electronic devices.

In addition to the above-mentioned honeycomb lattices, other two-dimensional arrangements of carbon, boron and nitrogen atoms have been suggested. To the best of our knowledge, these materials have been theoretically proposed and some of them have already been synthesized. Examples include graphynes,^25–29^ graphenylene,^30,31^ inorganic graphenylene (IGP),^32–34^ nitrogenated holey graphene (NHG),^35–37^ T-graphene,^38^ and others.^39–41^ First-principle calculations of structural optimization, phonon modes, and finite temperature molecular dynamics predict stability for all these structures. Investigations on these materials have also revealed promising mechanical, optical, and electronic properties for future technological applications. Among the cited examples, two of them have recently attracted a lot of attention and differ only by their chemical composition, graphenylene and IGP.

Graphenylene is a two-dimensional lattice of sp^2^-hybridized carbon atoms, which was first described by Balaban *et al.*^42^ This material is composed of six-membered rings (cyclohexatriene units) connected to four-membered rings, in a configuration with periodic pores of diameter 5.51 Å.^30,31^ This 2D structure is a semiconductor with a direct gap of 0.8 eV,^30,31^ which is promising for applications in semiconducting devices. Using simulations based on density functional theory, Brunetto *et al.*^31^ have shown that graphenylene can be formed spontaneously through a dehydrogenation process of porous graphene. Although the synthesis of graphenylene has not yet been achieved, porous graphene has already been obtained experimentally.^43^ Among the many possible applications of graphenylene, we point out the use of this structure as a molecular sieve: Song *et al.*^30^ and Zhu *et al.*^44^ have shown that graphenylene is a promising membrane for the separation of light gases, such as H_2_, N_2_, CO, CO_2_, and CH_4_, with remarkable high selectivity for H_2_. Hussain *et al.* has shown that graphenylene doped with alkali or alkaline earth metals, are promising materials for clean energy storage.^45^ Additionally, it was shown that graphenylene can be a good anode material for lithium-ion batteries.^46^

IGP, on the other hand, consists of B and N atoms arranged in a graphenylene lattice.^32–34^ Perim *et al.* performed *ab initio* calculations in order to investigate the structural and electronic properties of an IGP sheet.^32^ The authors found IGP to be stable in molecular dynamics simulations, and also found a cohesive energy only ∼6% smaller than that of h-BN. They also showed that, in the same way as graphenylene, IGP can be formed spontaneously through the dehydrogenation of porous BN (the BN analogue of porous graphene). IGP is an insulating material with large energy gap (≈4.10 eV), and possesses periodic pores of diameter 5.35 Å. DFT calculations performed by Xu *et al.* suggest that IGP could also be used as a molecular sieve for the separation of H_2_, N_2_, CO, and CO_2_ gases.^48^ Moreover, Perim *et al.*^32^ showed that the selective substitution of B and N atoms by C atoms in IGP significantly reduced the energy gap, changing its behavior from insulating to semiconducting. This result suggested the possibility of tuning the band gap of this material by changing its chemical composition.

**Fig. 1:**
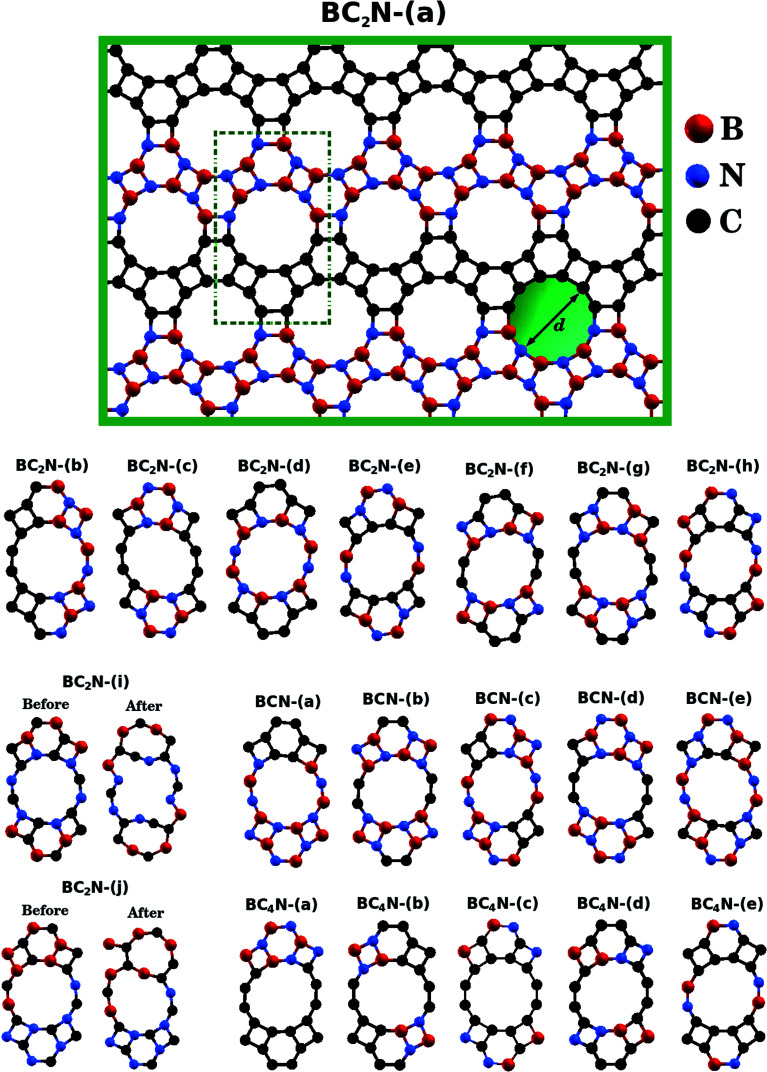
Illustration of the optimized B_*x*_C_*y*_N_*z*_ hybrid graphenylenes with different atomic arrangements.

Following the progress of investigations about honeycomb lattices, a natural development in the study of graphenylene monolayers is to consider the behavior of B_*x*_C_*y*_N_*z*_ hybrid nanosheets. So far, to the best of our knowledge, no theoretical or experimental studies exist in the literature that may have systematically investigated such lattices. Bearing this motivation in mind, and also considering the growing interest in B_*x*_C_*y*_N_*z*_ hybrid nanostructures, in the present contribution we employ first-principles calculations to investigate the structural stability and electronic properties of what we call B_*x*_C_*y*_N_*z*_ hybrid graphenylenes, shown in Fig. 1. In the structures considered here, rather than substituting selected atoms, we consider well-defined patterns mixing carbon, boron, and nitrogen atoms in a graphenylene geometry.

### 2. Computational details and methods

First principles calculations were performed using density functional theory (DFT) within the generalized gradient approximation (GGA) for the exchange-correlation term,^49,50^ as implemented in the SIESTA code.^51,52^ A linear combination of numerical atomic orbitals was used to represent a double-*ζ* basis set with polarized functions (DZP). To modulate the strong interactions between electrons and core ions, we used the norm-conserving Troullier Martins pseudopotential^53^ in the Kleinman-Bylander factorized form.^54^ In order to ensure an accurate description of the charge density in real space and calculate the self-consistent Hamiltonian matrix elements, we have utilized a mesh cutoff of 150 Ry. For the systems investigated, we have observed that a 6 × 4 × 1 special *k*-points grid is enough to give the correct geometry and stability. Self-consistency is achieved when the maximum difference between the output and the input of each element of the density matrix, in a self-consistent field cycle, is smaller than 10^−4^ eV. The optimization of atomic positions was allowed to proceed until the force on each atom was less than 0.05 eV Å^−1^. We adopted a rectangular supercell (Fig. 1) and all calculations were performed at *T* = 0 K. The unit cell is repeated in the *x* and *z* directions, forming an infinite B_*x*_C_*y*_N_*z*_ hybrid graphenylene sheet. A vacuum region of 20 Å was added along the *y*-axis to avoid artificial interaction between neighboring images.

In order to estimate the energetic stability of the B_*x*_C_*y*_N_*z*_ hybrid graphenylenes considered in this work, we calculate the formation energy *E*_Form_ through a thermodynamic approach based on the prior determination of the chemical potentials of the atomic species involved in the synthesis reaction. The details of this approach are described in ref. 15 and 16. The formation energy is defined by the following expression1
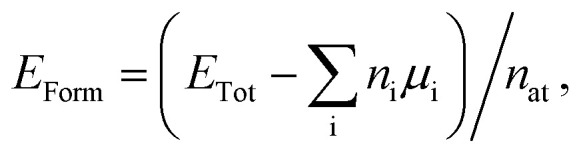
where *E*_Tot_ is the calculated total energy provided by the SIESTA code, *n*_i_ is the number of atoms for each element (i = B,N,C), *μ*_i_ is the corresponding chemical potential, and *n*_at_ is the total number of atoms in the structure. Moreover, the chemical potentials *μ*_B_, *μ*_N_, and *μ*_C_ must satisfy the conditions of thermodynamic equilibrium:

2 *μ*_BN_ = *μ*_B_ + *μ*_N_ and *μ*_CC_ = *μ*_C_ + *μ*_C_,

where the parameters *μ*_BN_ and *μ*_CC_ are the chemical potentials for the boron–nitrogen (BN) and carbon–carbon (CC) pairs, respectively. In this paper, the chemical potentials for the CC and BN pairs were obtained by taking graphenylene and IGP as references and assigning zero values to their formation energies. Thus, through the use of eqn (1) and (2), we found that *μ*_CC_ = −308.4 eV and *μ*_BN_ = −349.3 eV. Finally, for the hybrid graphenylenes studied in this work, we take into account that, in all cases, *n*_B_ = *n*_N_ = *n*_BN_, and *n*_CC_ = *n*_C_/2, so that eqn (1) can be rewritten as

3 *E*_Form_ = (*E*_Tot_ − *n*_BN_*μ*_BN_ − *n*_CC_*μ*_CC_)/*n*_at_.

## 3. Results and discussion

Fig. 1 shows the relaxed structure of the B_*x*_C_*y*_N_*z*_ hybrid graphenylenes with different stoichiometries (*x*, *y*, and *z*) investigated in this work. These structures are two-dimensional lattices of sp^2^-hybridized boron, carbon, and nitrogen atoms. They are formed by the fusion of four-membered and six-membered rings, resulting in a lattice with periodically distributed large pores. The nomenclature used to label the B_*x*_C_*y*_N_*z*_ graphenylenes refers to the minimal molecular formulas BCN, BC_2_N or BC_4_N. In order to construct the B_*x*_C_*y*_N_*z*_ graphenylenes, we considered a unit cell with 24 atoms (as indicated in Fig. 1 by a dashed rectangle) and different atomic arrangements. Thus, B_*x*_C_*y*_N_*z*_ graphenylenes have different numbers of B–N, C–C, B–C and N–C bonds. The chosen atomic arrangements preclude the presence of energetically unfavorable B–B and N–N bonds.^55^ Some interesting features presented by the considered structures include: (i) the presence of (BN)_*x*_ and C_*y*_ units of different sizes and forms; (ii) BN and C stripes connected along different directions; (iii) a high number of B–C and N–C bonds associated with a low number of B–N and C–C bonds (BC_2_N-(i) and BC_2_N-(j)).

In this context we point out that, before the DFT optimization, the unit cell of all B_*x*_C_*y*_N_*z*_ graphenylenes contained a circular pore of diameter *d*. However, our DFT optimization resulted in a substantial amount of structural stress, so that all final structures presented elliptical pores of varied shapes. In order to characterize the geometry of the pores, we measured the semi-major axis (*a*), the semi-minor axis (*b*), and calculated the eccentricity (*ε*) as4
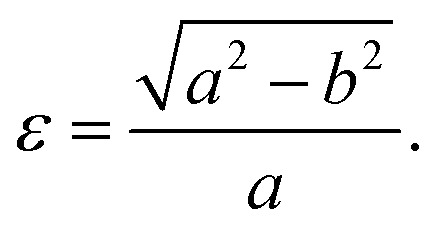


Values for 2*a*, 2*b*, and *c* are shown in Table 1. Out of all investigated structures, two (BC_2_N-(a), BC_2_N-(b)) retained pores of nearly circular shape (see Fig. 1). In these structures, each pore is half graphenylene and half IGP. Note that both graphenylene and IGP present circular pores. Conversely, notice that high eccentricity is strongly associated with multiple (BN)_*x*_ and C_*y*_ components dispersed through the unit cell. It is worth noting that some elliptical pores found by us are similar to the ellipsoidal cross sections of B_*x*_C_*y*_N_*z*_ hybrid nanotubes composed of diametrically opposed BN and C stripes, which were studied by Machado *et al.*^56^ and Guedes *et al.*^57^ On the other hand, for the BC_2_N-(i) and BC_2_N-(j) structures, which maximize the number of B–C and N–C bonds, we found extensive structural deformation. A two-dimensional lattice with non-graphenylene geometry arised as a result. From this, we conclude that maximizing the number of B–C and N–C bonds is not a favorable trend for B_*x*_C_*y*_N_*z*_ hybrid graphenylenes.

**Table 1:** Calculated formation energy (*E*_Form_) for all investigated B_*x*_C_*y*_N_*z*_ hybrid graphenylenes. *R*_b_ is the total number of ‘regular’ chemical bonds and *W*_b_ is the total number of ‘wrong’ chemical bonds. *E*_g_ is the energy band gap, 2*a* is twice the semi-major axis, 2*b* is twice the semi-minor axis, and *ε* is the eccentricity of pore. For a circle, 2*a* = 2*b* = *d*, where *d* is the diameter

Structure *x*	*E*_Form_ (eV/*n*_T_)	*R*_b_	*W*_b_	*R*_b_/*W*_b_	*E*_g_ (eV)	[2*a*][2*b*] (Å)	*ε*
BC_2_N-(a)	***0.08***	32	4	8.0	1.06	[5.61][5.51]	0.19
BC_2_N-(b)	0.15	28	8	3.5	0.16	[5.61][5.47]	0.22
BC_2_N-(c)	0.16	28	8	3.5	1.65	[5.76][5.02]	0.49
BC_2_N-(d)	0.17	28	8	3.5	0.53	[5.81][5.17]	0.46
BC_2_N-(e)	0.19	24	12	2.0	0.50	[5.54][5.24]	0.32
BC_2_N-(f)	0.21	24	12	2.0	1.02	[5.96][5.08]	0.52
BC_2_N-(g)	0.30	20	16	1.3	0.86	[5.92][5.21]	0.47
BC_2_N-(h)	0.32	20	16	1.3	0.75	[5.77][5.23]	0.42
BC_2_N-(i)	0.56	9	22	0.4	1.30	—	—
BC_2_N-(j)	0.79	4	30	0.1	0.00	—	—
BCN-(a)	***0.11***	30	6	5.0	1.09	[5.77][5.18]	0.44
BCN-(b)	0.17	28	8	3.5	0.77	[5.83][5.22]	0.45
BCN-(c)	0.17	28	8	3.5	0.51	[5.84][5.21]	0.45
BCN-(d)	0.17	28	8	3.5	1.10	[5.80][5.06]	0.49
BCN-(e)	0.25	20	16	1.3	1.05	[5.82][5.13]	0.47
BC_4_N-(a)	***0.11***	30	6	5.0	0.74	[5.80][5.09]	0.48
BC_4_N-(b)	0.17	28	8	3.5	0.36	[5.81][5.16]	0.46
BC_4_N-(c)	0.23	24	12	2.0	0.72	[5.60][5.39]	0.27
BC_4_N-(d)	0.26	20	16	1.3	0.38	[5.84][5.15]	0.47
BC_4_N-(e)	0.37	28	8	3.5	0.14	[5.83][5.22]	0.45

The results presented in Fig. 1 and Table 1 suggest a possible application for the B_*x*_C_*y*_N_*z*_ hybrid graphenylenes. Observe that the structures proposed present pores of diverse sizes, which are located in a range that is convenient for applications in gas separation. For instance, simulations have previously demonstrated that graphenylene membranes with pore size below 5.68 Å can separate H_2_ from CO_2_, CO, N_2_, and CH_4_ (in that investigation, pore size was controlled with strain).^44^ Other 2D membranes with diameters below this threshold have also displayed effective performance in H_2_ separation processes,^30,48,58,59^ further suggesting that hybrid graphenylenes could be used towards this end. Regarding the proposed structures with larger pores, some are located in a range that could be useful for the separation of different gases.^44^ Moreover, the varied pore shapes could prove useful in molecular sieves, as pores with roughly the same size and area can present different permeation ratios.^47^ Finally, further versatility could be attained by varying the polarity of the membrane, by selecting compositions with higher/lower fractions of B and N atoms. Polarity has been shown to influence both the selectivity and the permeance of IGP membranes; polarity may hinder or aid diffusion, depending on the gas composition.^48^ The great adaptability of hybrid graphenylenes suggests that particular pore sizes, shapes, and compositions could be selected to assemble sieves customized for varied gas separation tasks.

We also report in Table 1 the values of the formation energy for the B_*x*_C_*y*_N_*z*_ hybrid graphenylenes. Additionally, we note that there is a relationship between *E*_Form_ and the number, within the structure, of ‘regular’ chemical bonds, C–C and B–N, and ‘wrong’ chemical bonds, C–B and C–N. Thus, in order to quantify this effect, we define the ratio *R*_b_/*W*_b_, between the number of regular (*R*_b_) and wrong (*W*_b_) chemical bonds. The value of the ratio *R*_b_/*W*_b_, for each structure, is shown in Table 1. We notice an increase in the formation energy when the number of wrong bonds increases, *i.e.*, when the ratio *R*_b_/*W*_b_ decreases. This relationship has also been observed in other types of hybrid nanostructures with B_*x*_C_*y*_N_*z*_ stoichiometry.^15,16,60^

Bearing this in mind, notice in Table 1 that the BC_2_N-(a) graphenylene with the highest possible ratio *R*_b_/*W*_b_, presents the lowest *E*_Form_. Therefore, it is the most stable structure. Following this line of thought, the BCN-(a) and BC_4_N-(a) monolayers are tied as the second most stable structures, since they present the second lowest *E*_Form_ and the second highest *R*_b_/*W*_b_. Notice also that the difference between the *E*_form_ of these two structures and that of BC_2_N-(a) is only 0.03 eV/*n*_at_. The reason for the stability of these structures is found in their atomic arrangement, with BN and C stripes connected along the zigzag direction. In the case of the BC_2_N-(a) structure, where the width of the BN and C stripes are quite similar, we have a minimum amount of structural stress and a stable two-dimensional lattice. However, when the width of the BN and C stripes are different (BCN-(a) and BC_4_N-(a)), small deformations and pores with higher eccentricity appear in the lattice, slightly decreasing the stability. The most unstable structures are BC_2_N-(i) and BC_2_N-(j), which have the highest *E*_Form_ (over 0.5 eV/*n*_at_ above that of BC_2_N-(a)). Unsurprisingly, these configurations present the lowest *R*_b_/*W*_b_ ratio, maximizing the number of B–C and N–C bonds. They are also the only two that present optimized lattices that completely differ from the initial graphenylene geometry.

**Fig. 2:**
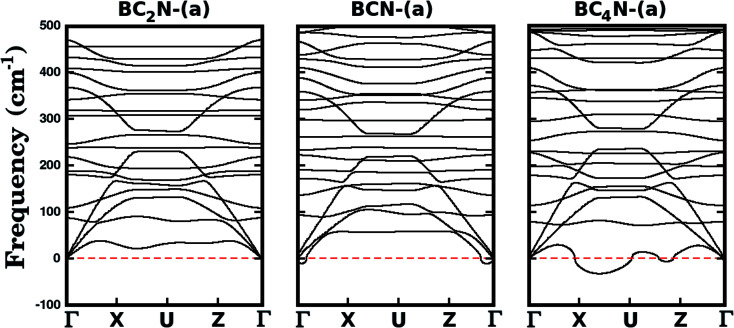
Phonon frequencies of selected B_*x*_C_*y*_N_*z*_ hybrid graphenylenes. The symmetry points are *Γ* = (0,0,0), *X* = (0.5,0,0), *U* = (0.5,0,0.5) and *Z* = (0,0,0.5) in reciprocal space coordinates, associated with a rectangular Brillouin zone. Imaginary frequencies are shown as negative values. The absence of negative frequencies in BC_2_N-(a) indicates its structural stability.

We further investigate the stability of the three most stable structures (BC_2_N-(a), BCN-(a), and BC_4_N-(a)) by calculating their phonon dispersion curves, presented in Fig. 2. Among the considered structures, BC_2_N-(a) is the only one that does not present imaginary frequencies (which are shown as negative values in Fig. 2), confirming its structural stability. Meanwhile, the presence of negative frequencies in the phonon dispersions of BCN-(a) and BC_4_N-(a) is an indication of possible structural instabilities. Furthermore, our prediction of structural stability based on the calculated phonon frequencies is in agreement with the calculated formation energies, with BC_2_N-(a) showing the lowest formation energy.

We also compare the formation energy of the BC_2_N-(a) graphenylene (which corresponds to the most stable structure) with the calculated formation energies for the B_*x*_C_*y*_N_*z*_ hexagonal monolayers,^14^ which have already been experimentally observed.^20,21^ In order to do so, we re-calculate the formation energy for the BC_2_N-(a) using the chemical potentials of the BN and CC pairs (*μ*_BN_ and *μ*_CC_) estimated for the B_*x*_C_*y*_N_*z*_ hexagonal monolayers, as done by Azevedo *et al.*^14^ The most stable hexagonal monolayer estimated by those authors is the one which presents stoichiometry B_3_C_2_N_3_, with formation energy of 0.12 eV/*n*_at_. For comparison purposes, we found that the BC_2_N-(a) graphenylene presents a formation energy 0.5 eV/*n*_at_ larger than B_3_C_2_N_3_.

**Fig. 3:**
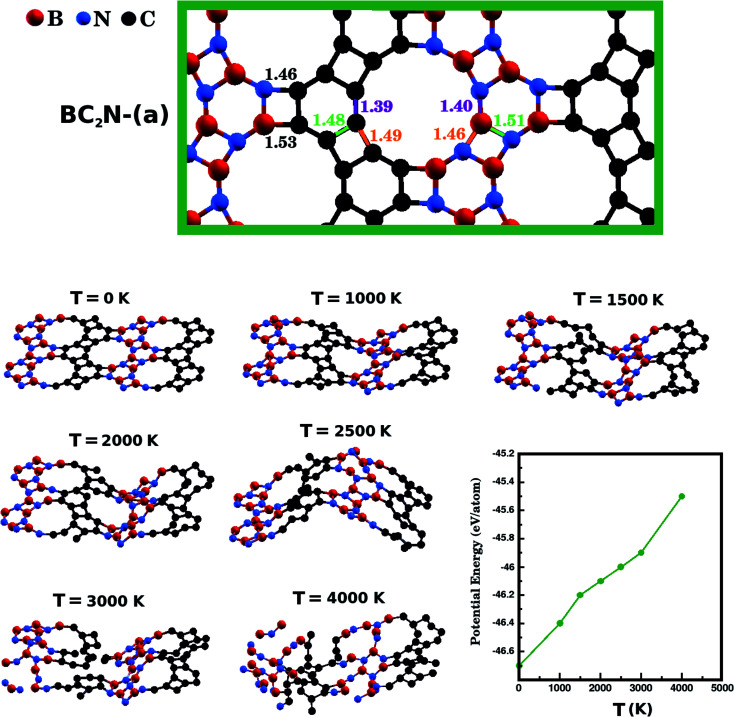
The optimized structure of BC_2_N-(a) and snapshots, from molecular dynamics simulations, of the optimized structure of BC_2_N-(a) after it was thermalized for 20 ps at different temperatures. Finally, the bottom-right plot displays the potential energy value after thermalization, for each of the investigated temperatures.

Next, we investigate the stability of the structure with lowest *E*_form_, BC_2_N-(a), performing *Ab Initio* Molecular Dynamics (AIMD) simulations using the SIESTA code. We used a time step of 1 fs to evolve BC_2_N-(a) for 20 ps in the NPT ensemble. We also employed the Nosé-Hoover thermostat and the Parrinello–Rahman barostat to control the temperature and in-plane pressure components, respectively. In order to avoid relaxation of the system in the direction perpendicular to the plane, we kept the monolayers separated by a vacuum slab of fixed length (20 Å). Finally, we performed AIMD stability tests at six temperatures: 1000 K, 1500 K, 2000 K, 2500 K, 3000 K, and 4000 K. In Fig. 3, we present snapshots of BC_2_N-(a) after thermalization, at different temperatures. We also present a plot relating the converged potential energy with temperature, which displays the proportionality between these two quantities. For 1000 K the thermalization process caused few changes in the atomic structure of BC_2_N-(a). From 1500 K to 2500 K, we observed that the graphenylene lattice remained intact, in spite of the increasing amplitude of the out-of-plane vibrations. From 3000 K onwards we observed bond breakage, and at 4000 K changes to the initial arrangement of bonds were extensive. In summary, our AIMD simulations indicate that the BC_2_N-(a) hybrid graphenylene is able to withstand high temperatures and is, therefore, very stable.

Additionally, Brunetto *et al.*^31^ and Perim *et al.*^32^ have shown that graphenylene and IGP can be formed spontaneously through a dehydrogenation process of porous graphene and porous BN, respectively. Although the synthesis of graphenylene has not yet been achieved, porous graphene has already been obtained experimentally.^43^ Thus, graphenylene could be obtained experimentally by using an electron beam as a means of selectively removing the hydrogen atoms from a porous graphene sheet, through the so-called knock-on effect,^61^ thus causing a spontaneous conversion to a stable graphenylene sheet. In view of this, we suggest that the B_*x*_C_*y*_N_*z*_ hybrid graphenylenes could also be obtained experimentally by removing the hydrogen atoms from a B_*x*_C_*y*_N_*z*_ porous graphene sheet by the knock-on effect. The synthesis of B_*x*_C_*y*_N_*z*_ porous graphene could be made using the same method for synthesis of B_*x*_C_*y*_N_*z*_ hybrid hexagonal monolayers.^20,21^

We have also calculated the B–N, C–C, B–C, and N–C bond lengths in the relaxed structures of the B_*x*_C_*y*_N_*z*_ hybrid graphenylenes. In the case of the most stable structure, BC_2_N-(a), we found three possible lengths for the C–C and B–N bonds. Specific values depend on the position in the lattice, since a bond can be part of a six-membered ring, of a four-membered ring, or can be shared by a six- and a four-membered ring (see Fig. 3). We measured C–C bond lengths of 1.39 Å, 1.49 Å, and 1.48 Å; and B–N bond lengths of 1.40 Å, 1.46 Å, and 1.51 Å, respectively. Similar results were also found in the literature for graphenylene and IGP, where, in both structures, there are three possible C–C and B–N bond lengths.^30,32^ The wrong bonds, B–C and N–C, measured ∼1.53 Å and 1.46 Å, respectively.

**Fig. 4:**
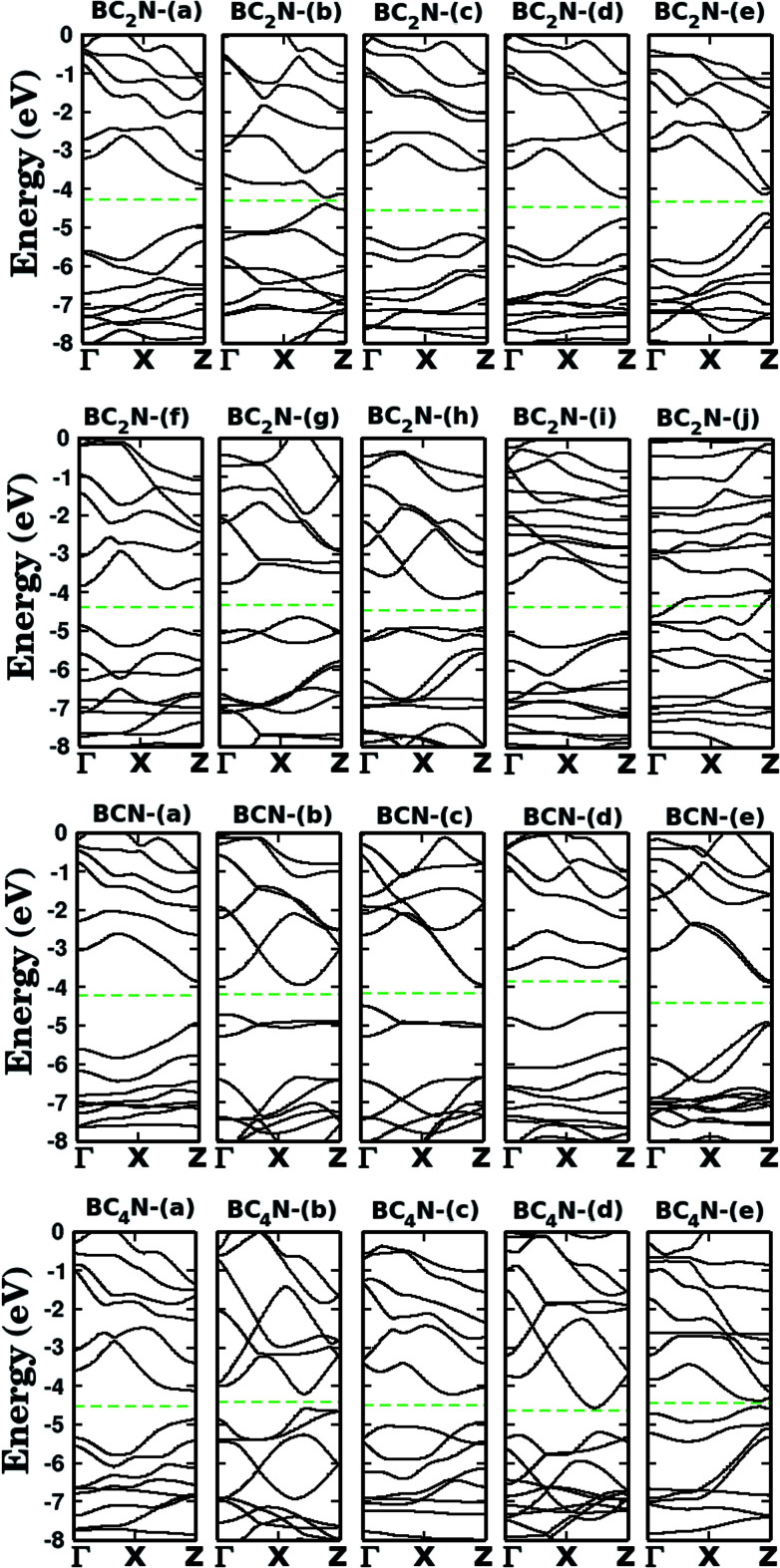
Calculated band structures for the monolayers shown in Fig. 1. The dashed line represents the Fermi level.

Let us now discuss the electronic properties of the B_*x*_C_*y*_N_*z*_ hybrid graphenylenes. In Fig. 4 we present the calculated electronic band structure with 100 *k*-points along the *Γ*–*X*–*Z* direction. The calculated energy band gaps *E*_g_ are shown in the fifth column of Table 1. We found *E*_g_ values ranging from 0.14 to 1.65 eV. With the exception of the BC_2_N-(i) and (j), which are not B_*x*_C_*y*_N_*z*_ hybrid graphenylenes, all the other structures behave as semiconductors, regardless of the atomic arrangement in the unit cell. It is interesting to note that the energy gap values of B_*x*_C_*y*_N_*z*_ graphenylenes oscillate above or below the estimated energy gap for pristine graphenylene (0.86 eV (ref. 31)), and are always lower than the estimated gap value for IGP (4.10 eV (ref. 32)). These results indicate that the inclusion of B and N atoms in the porous lattice of the graphenylene, for the formation of the B_*x*_C_*y*_N_*z*_ hybrid graphenylene, can increase or decrease the energy gap, but maintains the semiconducting behavior. This fact is associated with the presence of π-bonds and isolated electron pairs in the p_*z*_ orbitals of nitrogen atoms in the hexagonal rings. Moreover, the direct gap of 1.06 eV for the BC_2_N-(a) is about half of the estimated gap for the most stable B_*x*_C_*y*_N_*z*_ hexagonal monolayer, for which the gap is around 1.69 eV.^14,15^ Finally, we believe that our results for the energy gap of the B_*x*_C_*y*_N_*z*_ hybrid graphenylenes may open new perspectives for applications of these structures in future electronic nanodevices.

**Fig. 5:**
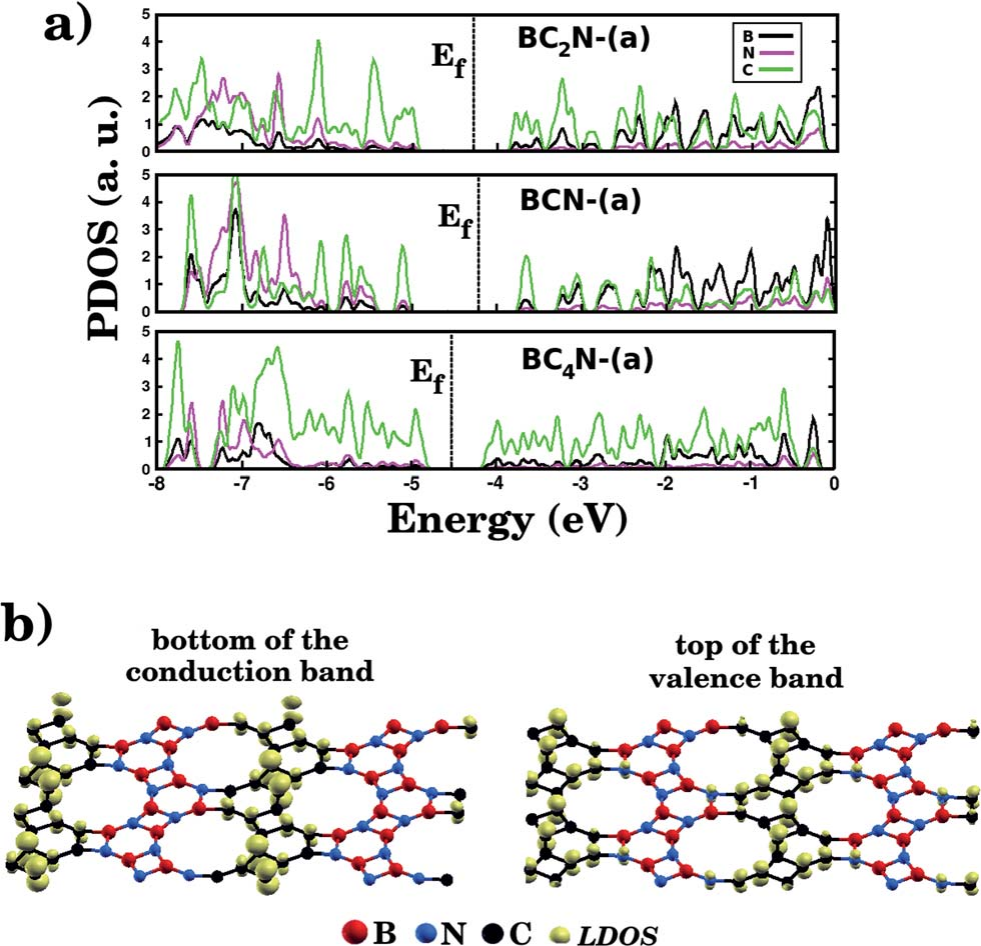
(a) The calculated projected density of states (PDOS) of selected B_*x*_C_*y*_N_*z*_ hybrid graphenylenes. The Fermi energy *E*_f_ is indicated by the dotted vertical line. (b) The local density of states (LDOS) associated with the bottom of the conduction band and the top of the valence band for the BC_2_N-(a) structure.

**Fig. 6:**
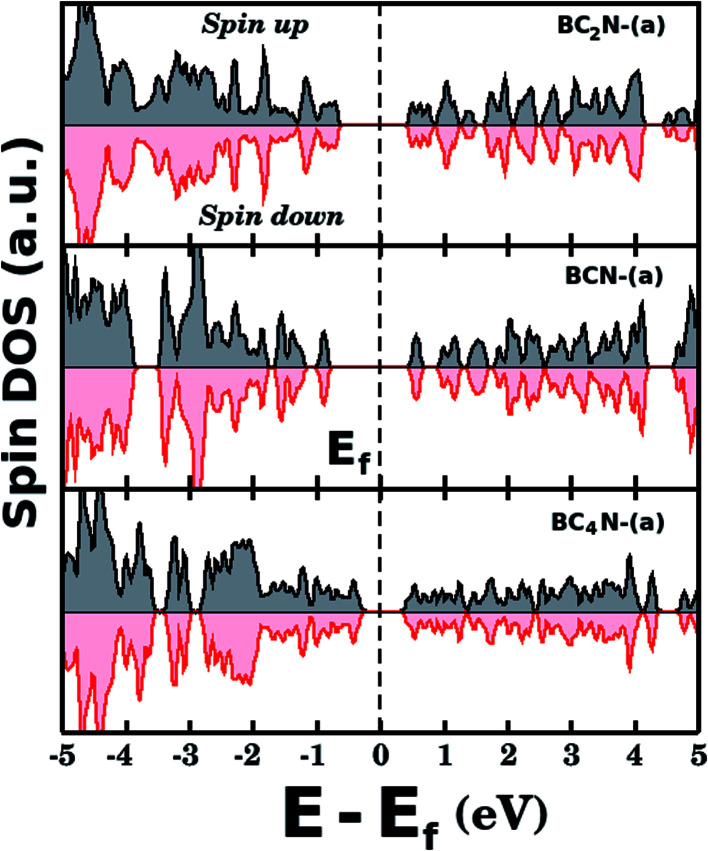
Densities of states (spin up and down) for selected B_*x*_C_*y*_N_*z*_ hybrid graphenylenes. The total spin is zero for all investigated structures.

Fig. 5(a) shows the projected density of states (PDOS) for selected B_*x*_C_*y*_N_*z*_ hybrid graphenylenes. We can infer from the PDOS that, in general, the contribution of the C atoms is more significant for electronic states near the Fermi energy *E*_f_. The contribution of the B atoms is stronger for electronic states in the conduction band, while the contribution of the N atoms is more significant in the valence band. This behavior is also observed in other types of nanostructures with stoichiometry B_*x*_C_*y*_N_*z*_, such as hexagonal monolayers,^23,24^ nanotubes^62,63^ and nanocones.^64^ Additional calculations of the localized density of states (LDOS) for the BC_2_N-(a) structure, shown in Fig. 5(b), reveal that the bottom of the conduction band and the top of the valence band are associated with the p_*z*_ orbitals of the C and N atoms, which is consistent with the results of the PDOS. For completeness, we show in Fig. 6 results of spin-polarization calculations, which reveal zero spin for all B_*x*_C_*y*_N_*z*_ hybrid graphenylenes structures, which means the valence band is completely filled and that unpaired electrons are not available.

**Fig. 7:**
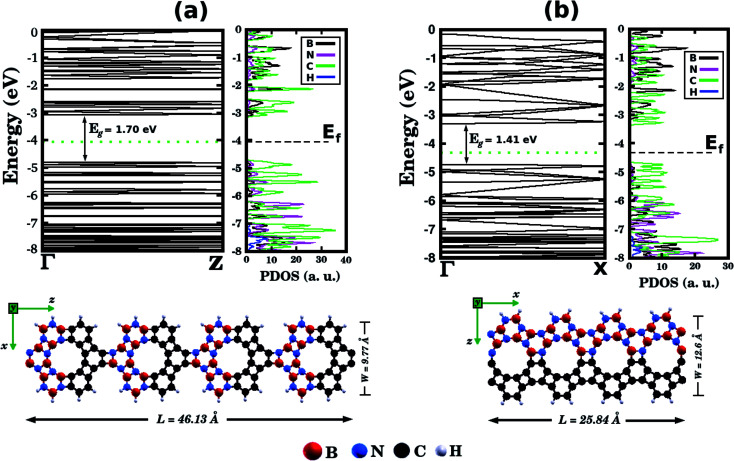
Illustrations of BC_2_N graphenylene nanoribbons with (a) armchair and (b) zigzag edges. The calculated band structures and the projected density of states (PDOS) are also shown. The Fermi energy *E*_f_ is indicated by the horizontal dotted line.

Before concluding, inspired by the recent work of Song *et al.*^30^ we performed first-principles calculations to probe electronic properties and structural stability of B_*x*_C_*y*_N_*z*_ graphenylene nanoribbons, as illustrated in Fig. 7. These structures were constructed based on the atomic arrangement of BC_2_N-(a), which is the most stable structure among all B_*x*_C_*y*_N_*z*_ hybrid graphenylenes. Thus, we considered nanoribbons composed of BN and C stripes, with either armchair (Fig. 7 (a)) or zigzag (Fig. 7(b)) symmetries. In particular, we notice that the BN and C stripes are perpendicular to the periodic direction in the armchair nanoribbon and parallel to the periodic direction in the zigzag nanoribbon. We found that the zigzag nanoribbon (total energy of −143.21 eV per atom) is more energetically favorable than the armchair one (total energy of −137.40 eV per atom). This result is associated with the fact that the zigzag nanoribbon presents a lower number of ‘wrong bonds’ (B–C and N–C) than the armchair one. Fig. 7 also shows the band structures of the studied nanoribbons. It can be seen that the zigzag nanoribbon exhibits a smaller energy gap *E*_g_ than the armchair one (a difference of 0.29 eV). However, both structures are semiconductors with an energy gap larger than that of the BC_2_N-(a) hybrid graphenylene. This result is similar to the one found for pristine graphenylene nanoribbons.^30^ Finally, from the corresponding PDOS shown in Fig. 7, it is possible to see that the electronic states near the Fermi level, which are responsible for the closing of the energy gap, are associated with carbon atoms.

## 4. Conclusions

In summary, we combined DFT calculations and AIMD simulations to investigate the stability and electronic properties of two-dimensional nanosheets, composed of B, C, and N atoms, with a geometry similar to that of graphenylene. We analyzed twenty structures with different atomic arrangements and stoichiometries, which we called B_*x*_C_*y*_N_*z*_ hybrid graphenylenes. We found that the most stable structure is the one with stoichiometry BC_2_N, which maximizes the number of B–N and C–C bonds, and minimizes the number of B–C and N–C bonds. This behavior is similar to that found for B_*x*_C_*y*_N_*z*_ hybrid honeycomb monolayers, for which the most stable structures also maximize the number of B–N and C–C bonds. Another interesting characteristic of the BC_2_N sheet is the atomic arrangement, with BN and C stripes connected along the zigzag direction. Regarding the structural properties, we found that the optimized structures of the B_*x*_C_*y*_N_*z*_ hybrid graphenylene present elliptical pores with different eccentricities and chemical compositions, which makes these structures particularly suited for molecular sieves. Our calculations also showed that the electronic properties of the B_*x*_C_*y*_N_*z*_ hybrid graphenylene are highly sensitive to the specific arrangement of B, C, and N atoms. We also found energy gaps ranging from 0.14 eV to 1.65 eV. Thus, all structures present semiconductor behavior, which may be interesting for the development of future electronic devices from these nanomaterials. Moreover, spin-polarized calculations demonstrated that none of the investigated structures exhibit magnetic behavior. Finally, we also investigated the electronic properties and the stability of B_*x*_C_*y*_N_*z*_ graphenylene nanoribbons. Our results show that the zigzag nanoribbon is more energetically favorable than the armchair one, and both present semiconductor behavior. We believe that this work adds an important group of monolayers to the ever increasing family of hybrid monolayers which combine B, C, and N atoms.

## Conflicts of interest

There are no conflicts to declare.

## Supplementary Material
